# Screening of the *TMEM151A* Gene in Patients With Paroxysmal Kinesigenic Dyskinesia and Other Movement Disorders

**DOI:** 10.3389/fneur.2022.865690

**Published:** 2022-05-30

**Authors:** Ling-Yan Ma, Lin Han, Meng Niu, Lu Chen, Ya-Zhen Yu, Tao Feng

**Affiliations:** ^1^Department of Neurology, Center for Movement Disorders, Beijing Tiantan Hospital, Capital Medical University, Beijing, China; ^2^China National Clinical Research Center for Neurological Diseases, Beijing, China; ^3^Running Gene Inc., Beijing, China; ^4^Department of Neurology, Hengshui Eighth People's Hospital, Hebei, China; ^5^Department of Encephalopathy, Dong Fang Hospital Affiliated to Beijing University of Chinese Medicine, Beijing, China; ^6^Department of Pediatrics, Beijing Tiantan Hospital, Capital Medical University, Beijing, China; ^7^Parkinson's Disease Center, Beijing Institute for Brain Disorders, Beijing, China

**Keywords:** paroxysmal kinesigenic dyskinesia (PKD), *TMEM151A* gene, dystonia, whole-exome sequencing (WES), paroxysmal exercise-induced dyskinesia (PED), paroxysmal non-kinesigenic dyskinesia (PNKD)

## Abstract

**Background:**

Paroxysmal kinesigenic dyskinesia (PKD) is a rare neurological disorder characterized by recurrent involuntary movements usually triggered by sudden movements. Mutations in the *TMEM151A* gene were found to be the causative factor of PKD in recent studies. It has also been revealed that loss-of-function is the mechanism by which *TMEM151A* mutations cause PKD.

**Methods:**

To investigate the genetic basis of PKD and broaden the clinical spectrum of the *TMEM151A* mutations, we recruited 181 patients of Chinese origin with movement disorders (MDs), including 39 *PRRT2*-negative PKD, 3 paroxysmal exercise-induced dyskinesia (PED), 2 paroxysmal non-kinesigenic dyskinesia (PNKD), 127 isolated dystonia, 8 choreas, and 2 myoclonus-dystonia syndromes. Whole-exome sequencing was applied to identify their possible disease-causing mutations. Then, Sanger sequencing was performed for validation and co-segregation analysis. Genetic analysis was also performed on additional family members of patients with *TMEM151A* mutations. Clinical manifestations of all PKD cases with mutations in *TMEM151A* reported, so far, were reviewed.

**Results:**

Two novel variants of the *TMEM151A* gene (NM_153266.4, NP_694998.1), c.627_643dup (p.A215Gfs^*^53) and c.627delG (p.L210Wfs^*^52), were identified in 2 patients with PKD by whole-exome sequencing and further Sanger sequencing. Both variants were inherited by the patients from their respective mothers. No mutation of the *TMEM151A* gene was found in the other type of movement disorders. In reviewing the clinical presentation of *TMEM151A*-related PKD, no statistically significant difference in the age of onset, family history, duration of attacks, laterality, and phenotype was found between genders. More male patients received treatment and had a good response. A higher proportion of female patients did not receive any treatment, possibly because they had a milder condition of the disease.

**Conclusions:**

This study further validated the role of *TMEM151A* in PKD. Future studies on protein function will be needed to ascertain the pathogenesis of *TMEM151A* in PKD.

## Introduction

Paroxysmal kinesigenic dyskinesia (PKD, OMIM_128200), also called episodic kinesigenic dyskinesia 1 (EKD1) or paroxysmal kinesigenic choreoathetosis (PKC), is a rare movement disorder characterized by recurrent attacks of involuntary movements triggered by sudden initiation or modification of movements (1). Short episodes include dystonia, chorea, or ballism and usually last less than a minute.

The PKD most commonly occurs sporadically and also occurs as an autosomal-dominant familial trait with varying penetrance. In 2011, the gene proline-rich transmembrane protein 2 (*PRRT2*) was first identified to be associated with PKD, which accounts for 77–93% familial and 21–45% isolated PKD (2, 3). Other genes, such as *PNKD* (paroxysmal nonkinesigenic dyskinesia protein), *SLC2A1* (solute carrier family 2 members 1), *KCNA1* (potassium voltage-gated channel subfamily A member 1), and *CHRNA4* (cholinergic receptor nicotinic alpha 4 subunits), have been proved to be associated with PKD as well (4–6). In recent studies, three research teams identified mutations of the *TMEM151A* gene (transmembrane protein 151A) as causative factors of PKD (7–9).

The heterogeneity of phenotypes and genotypes is common in different types of movement disorders. PKD usually manifests itself in various movement patterns, such as dystonia, chorea, and ballism. Previous studies showed that some causative genes of PKD can also be related to other diseases. Patients with mutations in *KCNA1* can present with PKD or Episodic Ataxia (10). *CHRNA4* was reported to be associated with insular epilepsy (11) and PKD. Thus, it is important to reveal whether the *TMEM151A* gene is related to other disorders. To further explore the role of *TMEM151A* mutations in PKD and other movement disorders and broaden the clinical and genetic spectrum of *TMEM151A* mutations, we performed whole-exome sequencing (WES) in 181 patients with different movement disorders. Two additional novel pathogenic variants were identified. Clinical presentations of *TMEM151A*-related patients with PKD was also reviewed.

## Materials and Methods

### Cohort Recruitment

We recruited 181 patients with various kinds of movement disorders, including 39 *PRRT2*-negative PKD, 3 paroxysmal exercise-induced dyskinesia (PED), and 2 paroxysmal non-kinesigenic dyskinesia (PNKD), 127 isolated dystonia, 8 chorea, and 2 myoclonus-dystonia syndromes. Of the 127 patients with isolated dystonia, 87 had focal dystonia (59 cervical dystonia, 17 blepharospasm, 4 oromadibular dystonia, 6 writer's cramp, and 1 spasmodic dysphonia), 12 had segmental dystonia, 7 had multifocal dystonia and 21 had generalized dystonia. All those who underwent WES also underwent a standardized neurological examination. All patients with PKD meet the diagnostic criteria for idiopathic PKD (12), including kinesigenic trigger for the attacks, a short duration of attacks (<1 min), lack of loss of consciousness or pain during attacks, a good response to antiepileptic drug treatment, exclusion of other organic diseases, and the onset age between 1 and 20 years. Other movement disorders were diagnosed according to their corresponding criteria (13, 14). After the study was approved by the Ethics Committee of the Beijing Tiantan Hospital, informed consents were obtained from all participants or their guardians.

### Whole-Exome Sequencing

Peripheral blood samples of patients and their family members were collected and sent to Running Gene Inc. (Beijing, China) for WES. Methods of WES have been described in previous research (15). Whole-exome sequencing was applied to all 181 patients to identify their possible disease-causing mutations. Sanger sequencing was then performed for validation and co-segregation analysis. Variants in *TMEM151A* gene were finally selected as candidate pathogenic mutations of PKD and other movement disorders.

## Results

### Clinical Presentation

**Figure 1:**
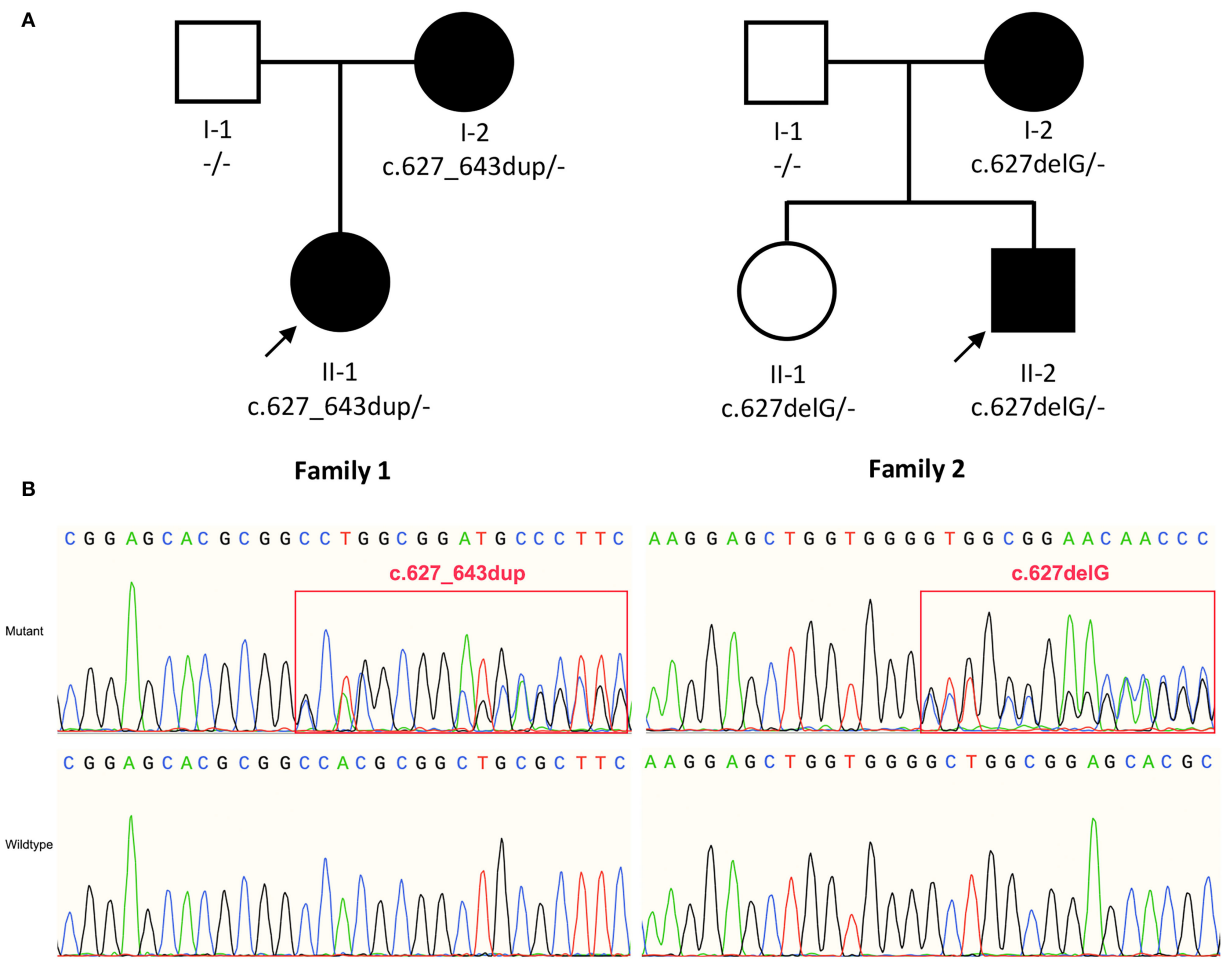
**(A)** Pedigree of family 1 and family 2. **(B)** Sanger sequencing show variant c.627_643dup (p. A215Gfs^*^53) and c.627delG (p.L210Wfs^*^52) identified in *TMEM151A* gene. The bold arrow indicates probands.

We identified variants of *TMEM151A* gene in two patients (Family 1 Patient II-1 and Family 2 Patient II-2, Figure 1A). No mutation of *TMEM151A* gene was found in other movement disorders.

Patient 1 was an 18-year-old woman, who has short attacks of dystonia in her left limb for 8 years. The involuntary movements were triggered by sudden activities after a period of physical rests and lasted ~10–30 s. Attack frequency was about 20 times per day. Occasionally, stress and startle can trigger her to have an episode. She was previously healthy without known significant abnormalities during her birth and growth. Her family history was not notable for involuntary movements, epilepsy, or other related diseases. This patient is the only child of her family, and her parents presented no neurological symptoms. Her treatment started at the age of 14 and her episodes were controlled satisfactorily with carbamazepine 200 mg per day. Six months ago (age 18), she tried to decrease the dose of carbamazepine, but her episodes' frequency has increased.

On physical examination, the vital signs were unremarkable and neurological examination was normal. Laboratory test results were within the normal range, including routine serum tests, standard biochemistry profile, hepatic and renal function, serum lactate concentration, ceruloplasmin, etc. Brain magnetic resonance imaging (MRI) and electroencephalogram (EEG) showed normal results.

Patient 2 was a 17-year-old man, who has short episodes of involuntary movement attacks in his both arms and left lower extremity for 8 years. The attacks were triggered by sudden voluntary movements and last about 30–60s. Both sides of his body could be involved, sometimes accompanied by occasional spasmodic torticollis. His consciousness was unaffected during the process. He had a full-term normal delivery and denied significant abnormalities during his birth growth. Carbamazepine was given at the age of 13 and his episodes showed a good response to carbamazepine 200 mg per day. During the past one year (age 16), he forgot to take the medicine several times and his episode frequency has increased. His mother had similar but very mild symptoms, presenting with short episodes of involuntary movement attacks in her both sides for <10 s. Her symptoms occurred occasionally for about 1 year and relieved completely with no treatment. His sister, who was born in 2000, carried the same mutation but without any symptoms.

On physical examination, his neurological signs were negative. The results of MRI scan were normal. There were no abnormities in his active electroencephalogram, electromyogram, electrocardiogram, blood routine test, thyroid function, liver and renal function, and serum electrolytes. Neurological examination of his sister was normal at both 4 years ago (age 18) and 5 months ago (age 21).

### Genetic Analysis

In our study, two novel variants of the *TMEM151A* gene (NM_153266.4, NP_694998.1), c.627_643dup (p. A215Gfs^*^53) and c.627delG (p.L210Wfs^*^52), were identified in patients with PKD by WES. Both variants were inherited by the patients from their respective mothers (Figure 1B).

Variant c.627_643dup (p. A215Gfs^*^53) is a small 17-bp duplication, leading to a frameshift of the subsequent amino acid sequences (remove >10% of protein) and a truncated product (PVS1_strong). This variant is absent from control databases, including ExAC v1.0 (16), gnomAD v2.1.1 (17), ESP6500SI-V2 (18), GenomeAsia 100K (19), and 1kGenomes v3.7.6 (20) (PM2_supporting). The variant co-segregated with PKD in this family (PP1). Multiple lines of *in silico* algorithms predicted c.627_643dup as deleterious, including MutationTaster 2021 (21) (deleterious), SIFT_indels (22) (damaging), FATHMM-indels (23) (score = 1, pathogenic), CADD v1.6 (24) (PHRED score = 32 > cutoff = 20, deleterious), MutPred-LOF (25) (score = 0.52 > cut-off = 0.50, possibly pathogenic), and CAPICE (26) (score = 0.54 > cutoff = 0.02, pathogenic) (PP3). Thus, according to the American College of Medical Genetics and Genomics (ACMG) guidelines (27), novel variant c.627_643dup (p.A215Gfs^*^53) is classified as “likely pathogenic” (PVS1_strong + PM2_supporting + PP1 + PP3).

Variant c.627delG (p. L210Wfs^*^52) is a 1-bp deletion that results in the frameshift causing more than 10% of protein missing, producing a strongly truncated proteins (PVS1_strong). This variant is absent from controls (PM2_supporting) and co-segregated with PKD in this family (PP1). Multiple *in silico* algorithms predicted c.627delG as deleterious, including MutationTaster 2021 (deleterious), SIFT_indels (damaging), FATHMM-indels (score = 0.99, pathogenic), CADD (PHRED score = 28.6 > 20, deleterious), MutPred-LOF (score = 0.52 >0.50, possibly pathogenic), and CAPICE (score = 0.58 > 0.02, pathogenic) (PP3). Therefore, novel variant c.627delG (p. L210Wfs^*^52) is also classified as “likely pathogenic” (PVS1_strong + PM2_supporting + PP1 + PP3).

It is noteworthy that patient II-1 (sister of patient II-2) in family 2 carries heterozygous c.627delG, but without showing any PKD-associated symptoms yet. We consider this as incomplete penetrance of *TMEM151A* mutations.

Apart from the *TMEM151A* gene, we also found several pathogenic mutations in other genes associated with other movement diseases. A heterozygous missense in *GNAL* (c.1060G>A, p.V354M) and a deletion (904_906/907_909del GAG) in *DYT1/TOR1A* were found separately in two patients with dystonia. Compound-heterozygous nonsense mutations in *VPS13A* (c.1162G>T, p.E388^*^ and c.2593C>T, p.R865^*^) were identified in one patient with chorea. A heterozygous insertion in *DYT11/SGCE* (c.658insGG, p. Glu220Glyfs^*^28) was found in one patient with dystonia-myoclonus syndrome.

## Discussion

Paroxysmal dyskinesias are a group of movement disorders characterized by episodes of involuntary movements (dystonia, chorea, athetosis, ballismus, or combined symptoms). They are classified into three main categories: paroxysmal kinesigenic dyskinesia (PKD), paroxysmal exercise-induced dyskinesia (PED), and paroxysmal non-kinesigenic dyskinesia (PNKD) (28). PKD, the most common type of paroxysmal dyskinesia, involves sudden attacks of dyskinesias induced by voluntary movements (28).

**Figure 2:**
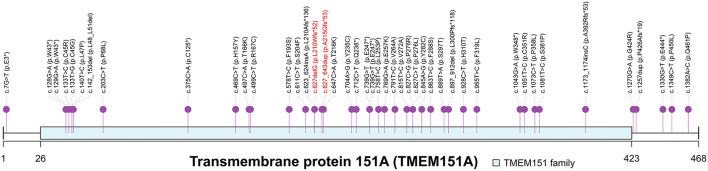
Spectrum of *TMEM151A* mutations. PKD-associated mutations of *TMEM151A* gene were pinned. Two novel variants were labeled in red. The asterisk symbol indicates frameshift mutation.

Gene *PRRT2* was first confirmed as the causative gene of PKD. However, the prevalence of *PRRT2* variants in patients with PKD ranges from 27 to 65% (2, 3), indicating that additional undiscovered causative genes are yet to be identified. With the help of next-generation sequencing, *TMEM151A* was identified as a causative gene for PKD (7–9). Li et al. (7) identified loss-of-function *TMEM151A* mutations, a frameshift and two missense variants, in three unrelated PKD families. They further screened *TMEM151A* variants in the WES data of 31 isolated Patients with PKD and found extra four truncated variants, three missense variants and a non-frameshift deletion in 8 isolated patients (25.8%, 8/31). Tian et al. (8) recruited a large cohort of patients with PKD and detected 24 heterozygous variants in *TMEM151A* in 25 probands (4.8%, 25/521), including 18 missense and 6 nonsense mutations, in which only one has been reported before. Li et al. (9) performed an exome-wide rare variant burden analysis in 86 PRRT2-negative PKD probands and identified 6 rare protein-altering *TMEM151A* variants (3 novel variants) in 10 unrelated probands. Chen et al. (29) also reported 7 variants (5 novel variants). So, a total of 44 *TMEM151A* mutations have been reported to be associated with PKD to date (Figure 2). Only mutations reported by Li et al. (7) have been recorded in Human Gene Mutation Database (HGMD) (30).

Currently, the function of *TMEM151A* is poorly understood. It is highly conserved among species. In mice, *Tmem151a* was highly expressed in the central nervous system (CNS), mainly in the cerebral cortex, hippocampus, spinal cord, brainstem, and thalamus (7). Until now, with our two patients added, 67 *TMEM151A*-related PKD cases were reported (7–9, 29) (see clinical manifestations of 67 cases in Supplementary Table). The mean age of onset of *TMEM151A*-related PKD is 11.99 ± 2.83 (mean ± SD), while male predominate (M: F = 2.53:1 [48:19]). Of these patients, 38.8% (26/67) had family history. There is no statistically significant difference in age of onset, family history, duration of attacks, laterality, and phenotype between genders (Table 1). When concerns to drug response, no statistical difference was found among patients with different drug reactions (complete relief, incomplete relief, and no treatment) (Table 2). However, a large percentage of female patients did not receive any treatments compared to their male counterparts, possibly because they had a mild condition. More male patients received treatment and regained their health.

**Table 1:** Clinical features in male and female cases.

	**Male**	**Female**	***p* value**
	**(*n* = 48)**	**(*n* = 19)**	
**Mean age at onset (years)**	11.98 ± 2.96	12.00 ± 2.52	0.979
**With family history (n) (%)**	17 (35.4)	9 (47.4)	0.366
**Episode duration**			0.187
** <10 s (n) (%)**	31 (64.6)	16 (84.2)
**>10 s (n) (%)**	17 (35.4)	3 (15.8)
**Phenotype**			0.472
**Dystonia (n) (%)**	28 (58.3)	12 (63.2)
**Chorea (n) (%)**	11 (22.9)	2 (10.5)
Dystonia plus other forms*(n) (%)	9 (18.8)	5 (26.3)
**Laterality**			0.284
**Unilateral (n) (%)**	16 (33.3)	9 (47.4)
**Bilateral involved (n) (%)**^**#**^	32 (66.7)	10 (52.6)
**Response to drug**			0.021
**Complete (n) (%)**	27 (56.3)	6 (31.6)
**Incomplete (n) (%)**	12 (25.0)	3 (15.8)
**No treatment**	9 (18.8)	10 (52.6)

*Dystonia plus with chorea or ballism; ^#^Bilateral or bilateral alternation.

**Table 2:** Differences between in different drug reactivity groups.

	**Complete** **(*n =* 33)**	**Incomplete** **(*n =* 15)**	**No treatment** **(*n =* 19)**	**p-value**
**Mean age at onset (years)**	12.03 ± 2.90	11.20 ± 2.46	12.53 ± 2.97	0.400
**Male (** ***n*** **) (%)**	27 (81.8)	12 (80.0)	9 (47.4)	0.021
**With family history (** ***n*** **) (%)**	9 (27.3)	5 (33.3)	12 (63.2)	0.034
**Episode duration**				0.478
** <10s (*****n*****) (%)**	21 (63.6)	12 (80.0)	14 (73.7)	
**>10s (*****n*****) (%)**	12 (36.4)	3 (20.0)	5 (26.3)	
**Phenotype**				0.582
**Dystonia (*****n*****) (%)**	18 (54.5)	9 (60.0)	13 (68.4)	
**Chorea (*****n*****) (%)**	9 (27.3)	2 (13.3)	2 (10.5)	
Dystonia plus other forms*(***n*****) (%)**	6 (18.2)	4 (26.7)	4 (21.1)	

*Dystonia plus with chorea or ballism.

As previously reported in the literature, no significant differences were found between *PRRT2*-positive and *TMEM151A*-positive PKD in terms of attack type and treatment outcome (8). However, patients with *TMEM151A* mutations were prone to have a later-onset age, shorter attack durations, and residual attacks/aura when treated with carbamazepine or oxcarbazepine (8, 29).

In this study, we identified two novel variants of the *TMEM151A* gene in two patients with PKD and summarized the clinical and genetic features of all patients reported, so far. We did not find any correlation between the *TMEM151A* gene and other movement disorders. Further studies on the function of the *TMEM151A* gene and protein are expected to determine the pathogenesis of PKD in the future.

## Data Availability Statement

The datasets presented in this study can be found in online repositories. The name of the repository and accession number can be found below: Genome Sequence Archive for Human (GSA-Human) in the National Genomics Data Center (NGDC), China National Center for Bioinformation (CNCB)/Beijing Institute of Genomics (BIG), Chinese Academy of Sciences (CAS), https://ngdc.cncb.ac.cn/gsa-human/, HRA001662.

## Ethics Statement

The studies involving human participants were reviewed and approved by Beijing Tiantan Hospital. The patients/participants provided their written informed consent to participate in this study. Written informed consent was obtained from the individual(s) for the publication of any potentially identifiable images or data included in this article.

## Author Contributions

L-YM participated in the design of the study and drafted the manuscript. LH interpreted genetic data and contributed to the manuscript. TF and Y-ZY carried out the conceptualization of the study, reviewing, and critiquing the article at the same time. MN and LC collected the medical data of the patients. All authors have read and approved the final manuscript.

## Funding

This research was supported by the Natural Science Foundation of China (Nos. 82071422 and 81571226) and Beijing Natural Science Foundation (Nos. 7164254 and 7212031).

## Conflict of Interest

LH was employed by Running Gene Inc. Beijing, China. The remaining authors declare that the research was conducted in the absence of any commercial or financial relationships that could be construed as a potential conflict of interest.

## Publisher's Note

All claims expressed in this article are solely those of the authors and do not necessarily represent those of their affiliated organizations, or those of the publisher, the editors and the reviewers. Any product that may be evaluated in this article, or claim that may be made by its manufacturer, is not guaranteed or endorsed by the publisher.
