# Supplementary material for: Screening of the TMEM151A Gene in Patients With Paroxysmal Kinesigenic Dyskinesia and Other Movement Disorders
Source: Front Neurol. 2022 May 30;13:865690. doi: 10.3389/fneur.2022.865690 (PMC9189402; doi:10.3389/fneur.2022.865690)
Supplement: Supplementary file 1 [file Table_1.docx]

| **Patient/gender**  **Table**. Clinical and genetic features of *TMEM151A*-associated PKD cases | **Gender** | **Onset age (year)** | **Family History** | **Duration (second)** | **Phenotype** | **Laterality** | **Response to drug** | **Variant** |
| --- | --- | --- | --- | --- | --- | --- | --- | --- |
| 24/M^[1]^ | M | 6 | No | 10-30 | D | Unilateral or bilateral | Complete | c.845A>G (p.Tyr282Cys) |
| 18/M^[1]^ | M | 8 | No | 10-30 | D | Bilateral | Complete | c.1043G>A (p.Trp348*) |
| 16/M^[1]^ | M | 8 | No | 10-30 | D | Unilateral | No treatment | c.769G>A (p.Glu257Lys) |
| Family 1 (III-2)/M^[2]^ | M | 9 | Yes | <10 | D | Bilateral | Incomplete | c.1275dupG（p.P426Afs*19） |
| Isolated case 1/F^[2]^ | F | 9 | No | <10 | D/C | Bilateral | Incomplete | c.7G>T（p.E3*） |
| Isolated case 2/F^[2]^ | F | 9 | No | <10 | D/C | Bilateral | Complete | c.140 T>C（p.L47P） |
| 20/M^[1]^ | M | 9 | No | 10-30 | D & C | Bilateral | Complete | c.133T>G (p.Cys45Gly) |
| 12/M^[1]^ | M | 9 | Yes | <10 | D | Unilateral | No treatment | c.791T>C (p.Val264Ala) |
| Family 1 (II-5)/M^[2]^ | M | 10 | Yes | <10 | D/E | Unilateral | Incomplete | c.1275dupG（p.P426Afs*19） |
| Family 2 (II-4)/F^[2]^ | F | 10 | Yes | <10 | D | Bilateral | No treatment | c.375C>A（p.C125*） |
| Family 3 (II-1)/M^[2]^ | M | 10 | Yes | <10 | D | Unilateral | No treatment | c.758T>C（p.L253P） |
| Family 3 (III-2)/F^[2]^ | F | 10 | Yes | <10 | D | Unilateral | Incomplete | c.758T>C（p.L253P） |
| 26/M^[1]^ | M | 10 | No | <10 | D | Bilateral | No treatment | c.1081T>C (p.Ser361Pro) |
| 22/F^[1]^ | F | 10 | Yes | <10 | D & Ballism | Unilateral | No treatment | c.499C>T (p.Arg167Cys) |
| Family 2 (III-1)/F^[2]^ | F | 11 | Yes | <10 | D | Unilateral | No treatment | c.375C>A（p.C125*） |
| Family 2 (III-2)/F^[2]^ | F | 11 | Yes | <10 | D/E | Bilateral | Complete | c.375C>A（p.C125*） |
| Isolated case 7/M^[2]^ | M | 11 | No | <10 | D | Bilateral | Incomplete | c.889T>A（p.S297T） |
| 22/F^[1]^ | F | 11 | Yes | <10 | D | Unilateral | No treatment | c.499C>T (p.Arg167Cys) |
| Family 1 (III-1)/M^[2]^ | M | 12 | Yes | <10 | D | Bilateral | Incomplete | c.1275dupG（p.P426Afs*19） |
| Isolated case 3/M^[2]^ | M | 12 | No | <10 | D | Bilateral | Incomplete | c.142_153del（p.48_51del） |
| Isolated case 5/M^[2]^ | M | 12 | No | <10 | D | Bilateral | Incomplete | c.739G>T（p.E247*） |
| Isolated case 8/M^[2]^ | M | 12 | No | <10 | D | Bilateral | Complete | c.897_912del（p.L300Pfs*118） |
| 30/F^[1]^ | F | 12 | No | <10 | D | Unilateral | Complete | c.1051T>C(p.Cys351Arg) |
| 28/M^[1]^ | M | 12 | No | <10 | D, C & Ballism | Unilateral or bilateral | Complete | c.375C>A (p.Cys125*) |
| 26/M^[1]^ | M | 12 | No | <10 | D | Unilateral or bilateral | Complete | c.497C>A (p.Thr166Lys) |
| 15/M^[1]^ | M | 12 | No | <10 | D | Unilateral | No treatment | c.955T>C (p.Phe319Leu) |
| 22/M^[1]^ | M | 12 | Yes | <10 | D | Bilateral alternation | Complete | c.578T>C (p.Phe193Ser) |
| Isolated case 6/M^[2]^ | M | 13 | No | <10 | C | Bilateral | Complete | c.863T>C（p.F288S） |
| 22/F^[1]^ | F | 13 | No | <10 | D | Unilateral or bilateral | Complete | c.1073C>T (p.Pro358Leu) |
| 15/F^[1]^ | F | 13 | No | 10-30 | D | Bilateral | No treatment | c.129G>A (p.Trp43*) |
| 13/M^[1]^ | M | 13 | Yes | <10 | D & C | Bilateral | Complete | c.704A>G (p.Tyr235Cys) |
| 15/M^[1]^ | M | 13 | No | 30-60 | D & C | Bilateral | Incomplete | c.815T>C (p.Val272Ala) |
| Isolated case 4/M^[2]^ | M | 14 | No | <10 | D | Unilateral | Complete | c.623_624insA（p.L210Afs*136） |
| 25/M^[1]^ | M | 14 | Yes | 10-30 | D & C | Unilateral or bilateral | No treatment | c.203C>T (p.Pro68Leu) |
| Family 1 (II-3)/M^[2]^ | M | 15 | Yes | <10 | D | Unilateral | No treatment | c.1275dupG（p.P426Afs*19） |
| 18/F^[1]^ | F | 15 | No | <10 | D | Unilateral | No treatment | c.827C>G (p.Pro276Arg) |
| 21/F^[1]^ | F | 15 | No | 10-30 | D | Unilateral or bilateral | Incomplete | c.928C>T (p.His310Tyr) |
| 26/M^[1]^ | M | 15 | No | <10 | D | Bilateral | Complete | c.712C>T (p.Gln238*) |
| 15/M^[1]^ | M | 15 | Yes | <10 | D | Bilateral | Complete | c.704A>G (p.Tyr235Cys) |
| 22/M^[1]^ | M | 16 | No | <10 | D & C | Unilateral or bilateral | Incomplete | c.133T>C (p.Cys45Arg) |
| 23/M^[1]^ | M | 16 | Yes | 10-30 | D & C | Bilateral | Complete | c.128G>A (p.Trp43*) |
| 24/M^[1]^ | M | 16 | Yes | 10-30 | D | Bilateral | Complete | c.791T>C (p.Val264Ala) |
| 23/M^[1]^ | M | 16 | No | 10-30 | D | Unilateral | No treatment | c.375C>A (p.Cys125*) |
| 49/M^[1]^ | M | 17 | Yes | 10-30 | D & C | Bilateral alternation | No treatment | c.203C>T (p.Pro68Leu) |
| 29/F^[1]^ | F | 18 | No | <10 | D & Ballism | Unilateral or bilateral | No treatment | c.827C>T (p.Pro276Leu) |
| 25/M^[1]^ | M | 19 | No | <10 | D | Unilateral | Complete | c.1330G>T (p.Glu444*) |
| Patient 1 | F | 10 | No | 10-30 | D | Unilateral | Complete | c.627_643dup (p.A215Gfs*53) |
| Patient 2 | M | 9 | Yes | 30-60 | D | Bilateral | Complete | c.627delG (p.L210Wfs*52) |
| PKD22-1^[3]^ | M | 13 | No | <10s | D | Bilateral alternation | Complete | c.897_912delCCTGCGCGTCGTGGCC（p.L300Pfs*118） |
| PKD32-1^[3]^ | M | 12 | No | <10s | C | Unilateral | Incomplete | c.897_912delCCTGCGCGTCGTGGCC（p.L300Pfs*118） |
| PKD48-1^[3]^ | M | 14 | No | <10s | C | Bilateral alternation | Complete | c.375C > A（p.C125*） |
| PKD91-1^[3]^ | M | 7 | No | <10s | C | Bilateral alternation | Complete | c.897_912delCCTGCGCGTCGTGGCC（p.L300Pfs*118） |
| PKD99-1^[3]^ | M | 12 | No | 10-30s | C | Unilateral | Complete | c.611C > T（p.S204F） |
| PKD113-1^[3]^ | M | 12 | No | 10-30s | D | Unilateral | Complete | c.1173_1174insC（p.A392Rfs*53） |
| PKD126-1^[3]^ | M | 13 | Yes | <10s | D | Unilateral | Complete | c.1349C > T（p.P450L） |
| PKD126-M^[3]^ | F | 13 | Yes | <10s | D | Unilateral | No treatment | c.1349C > T（p.P450L） |
| PKD128-1^[3]^ | M | 13 | Yes | <10s | C | Unilateral | Complete | c.897_912delCCTGCGCGTCGTGGCC（p.L300Pfs*118） |
| PKD128-M^[3]^ | F | 16 | Yes | <10s | C | Unilateral | No treatment | c.897_912delCCTGCGCGTCGTGGCC（p.L300Pfs*118） |
| PKD131-1^[3]^ | M | 15 | No | 10-30s | C | Unilateral | Complete | c.791 T > C（p.V264A） |
| PKD132-1^[3]^ | M | 13 | No | 10-30s | C | Bilateral | Complete | c.897_912delCCTGCGCGTCGTGGCC（p.L300Pfs*118） |
| Family 1 (III-1)^[4]^ | M | 7 | Yes | <10s | C | Bilateral | Incomplete | c.[469C>T];[1382A>C] |
| Family 1 (II-2)^[4]^ | F | 10 | Yes | <10s | C | Bilateral | No treatment | c.469C>T（p.H157Y） |
| Sporadic case 1 ^[4]^ | M | 11 | No | <10s | C | Bilateral | Complete | c.140 T>C（p.L47P） |
| Sporadic case 2^[4]^ | F | 12 | No | <10s | D? | Bilateral | Complete | c.647C>A（p.T216K） |
| Sporadic case 3^[4]^ | M | 8 | No | 10-30s | D | Bilateral | Incomplete | c.739G>T（p.E247*） |
| Sporadic case 4^[4]^ | M | 6 | No | 10-30s | C | Bilateral | Complete | c.827C>T（p.P276L） |
| Sporadic case 5^[4]^ | M | 12 | No | <10s | D | Unilateral | Incomplete | c.1270G>A（p.G424R） |

D：dystonia；C：chorea; M：male；F：female

**References**

[1] Tian WT, Zhan FX, Liu ZH, et al. TMEM151A Variants Cause Paroxysmal Kinesigenic Dyskinesia: A Large-Sample Study. Mov Disord. 2021 .

[2] Li HF, Chen YL, Zhuang L, et al. TMEM151A variants cause paroxysmal kinesigenic dyskinesia. Cell Discov. 2021. 7(1): 83.

[3] Li YL, Lv WQ, Zeng YH, et al. Exome-Wide Analyses in Paroxysmal Kinesigenic Dyskinesia Confirm TMEM151A as a Novel Causative Gene. Mov Disord. 2022. 37(3): 641-643.

[4] Chen YL, Chen DF, Li HF, Wu ZY. Features Differ Between Paroxysmal Kinesigenic Dyskinesia Patients with PRRT2 and TMEM151A Variants. Mov Disord. 2022. 37(3): 608-613.
